# Modulation of the human gut microbiota by dietary fibres occurs at the species level

**DOI:** 10.1186/s12915-015-0224-3

**Published:** 2016-01-11

**Authors:** Wing Sun Faith Chung, Alan W. Walker, Petra Louis, Julian Parkhill, Joan Vermeiren, Douwina Bosscher, Sylvia H. Duncan, Harry J. Flint

**Affiliations:** Microbiology Group, Rowett Institute of Nutrition and Health, University of Aberdeen, Greenburn Road, Bucksburn, Aberdeen, Scotland AB21 9SB UK; Pathogen Genomics Group, Wellcome Trust Sanger Institute, Hinxton, Cambridgeshire, CB10 1SA UK; Cargill R&D Centre Europe, Vilvoorde, Belgium

**Keywords:** Bacteroidetes, Prebiotic, Colonic anaerobes, *Faecalibacterium prausnitzii*, Firmicutes, Inulin, Pectin, Propionate

## Abstract

**Background:**

Dietary intake of specific non-digestible carbohydrates (including prebiotics) is increasingly seen as a highly effective approach for manipulating the composition and activities of the human gut microbiota to benefit health. Nevertheless, surprisingly little is known about the global response of the microbial community to particular carbohydrates. Recent *in vivo* dietary studies have demonstrated that the species composition of the human faecal microbiota is influenced by dietary intake. There is now potential to gain insights into the mechanisms involved by using *in vitro* systems that produce highly controlled conditions of pH and substrate supply.

**Results:**

We supplied two alternative non-digestible polysaccharides as energy sources to three different human gut microbial communities in anaerobic, pH-controlled continuous-flow fermentors. Community analysis showed that supply of apple pectin or inulin resulted in the highly specific enrichment of particular bacterial operational taxonomic units (OTUs; based on 16S rRNA gene sequences). Of the eight most abundant *Bacteroides* OTUs detected, two were promoted specifically by inulin and six by pectin. Among the Firmicutes, *Eubacterium eligens* in particular was strongly promoted by pectin, while several species were stimulated by inulin. Responses were influenced by pH, which was stepped up, and down, between 5.5, 6.0, 6.4 and 6.9 in parallel vessels within each experiment. In particular, several experiments involving downshifts to pH 5.5 resulted in *Faecalibacterium prausnitzii* replacing *Bacteroides* spp. as the dominant sequences observed. Community diversity was greater in the pectin-fed than in the inulin-fed fermentors, presumably reflecting the differing complexity of the two substrates.

**Conclusions:**

We have shown that particular non-digestible dietary carbohydrates have enormous potential for modifying the gut microbiota, but these modifications occur at the level of individual strains and species and are not easily predicted a priori. Furthermore, the gut environment, especially pH, plays a key role in determining the outcome of interspecies competition. This makes it crucial to put greater effort into identifying the range of bacteria that may be stimulated by a given prebiotic approach. Both for reasons of efficacy and of safety, the development of prebiotics intended to benefit human health has to take account of the highly individual species profiles that may result.

**Electronic supplementary material:**

The online version of this article (doi:10.1186/s12915-015-0224-3) contains supplementary material, which is available to authorized users.

## Background

The human large intestine harbours up to 10^14^ bacteria, whose combined degradative and biochemical capabilities greatly exceed those of their host. The interplay between the host and its gut microbiota, via degradative activities that yield nutrients and metabolites and via interactions with the immune system, is highly complex, but the consequences for human health are increasingly recognised. Alterations in the gut environment and imbalances of intestinal homeostasis are associated with changes in microbiota composition during the development of gastrointestinal diseases [1], while microbiota composition is also thought to influence energy recovery from the diet, metabolic regulation, hormone signalling and systemic health [2, 3].

Although there is considerable variation in the gut microbiota even between healthy individuals, volunteer studies involving carefully controlled diets have now established that dietary intake exerts an important influence on the composition of the human gut microbiota [4–7]. These changes may reflect selective growth promotion by dietary components that provide the main energy sources for gut bacteria, as well as inhibitory effects resulting in particular from lipid intake and bile metabolism [5]. Human diets provide energy sources available to the large intestinal microbiota mainly in the form of non-digestible (ND) carbohydrates (plant fibre and resistant starch). Studies in which ND carbohydrate intake was varied in diets with matching macronutrient composition have established that the type of ND carbohydrate can alter microbiota composition, and that such changes occur within a few days [4]. Prebiotics based on ND carbohydrates, especially inulin and fructo-oligosaccharides, are already widely used with the aim of stimulating bacterial species and activities that are considered beneficial to health among the resident microbiota [8]. Most studies on prebiotics have focussed on selected target groups rather than the whole microbial community, but these also provide evidence for the selective stimulation of certain groups or genera within the human gut microbiota [9, 10–11].

It is not possible to establish from *in vivo* studies whether changes in microbiota composition resulting from dietary supplementation with a ND carbohydrate are due to direct stimulation of growth by the substrate, or alternatively to indirect effects such as the acidification of the colon that follows the ingestion of any fermentable fibre due to subsequent generation of short chain fatty acids (SCFAs) by the microbiota [11, 12]. This question can, however, be answered *in vitro* by using anaerobic pH-controlled continuous flow fermentors inoculated with human faecal microbiota. In previous work, we showed that, with ND polysaccharides (mainly starch) as a growth substrate, a one unit pH shift caused a major change in composition of the human colonic microbiota, with Gram-negative *Bacteroides* species predominating at pH 6.5, but Gram-positive Firmicutes increasing at pH 5.5 [11, 13]. We decided to use this approach to determine to what extent different ND carbohydrates affect the composition of the gut microbiota at a given controlled pH. Specifically, the experiments reported here compare the impact of inulin and pectin upon the microbiota. Inulin is a plant storage polysaccharide that consists of linear chains of fructose residues with a β-(2–1) linkage and is the basis for many existing prebiotics. Pectin comprises a highly complex set of plant cell wall polysaccharides that includes homogalacturonan and rhamnogalacturonan I and II, with side chains of arabinans, galactans, and arabinogalactans [14]. Our results show that inulin and pectin exert highly selective effects upon the gut microbiota not at the phylum level, but at the level of individual species, with very little overlap between the two substrates in the species promoted. Since pH is an important factor governing the competition between bacterial species [13] and luminal pH differs between the proximal and distal colon, it was important to obtain information across the physiologically relevant pH range. Responses are shown to be influenced quantitatively by the pH across the range 5.5–6.9. These findings have important consequences for our understanding of nutritional specialisation among human colonic bacteria and for predicting how diet composition, including the addition of prebiotics, can be used to manipulate microbiota composition.

## Results

### Influence of pH on competition for inulin and apple pectin in continuous culture

**Fig. 1:**
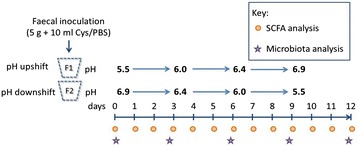
Diagram showing the design of the fermentor experiments used in this study. Single substrates (inulin or apple pectin) were supplied at a concentration of 0.5 %. In each experiment, two vessels were run in parallel (F1 and F2), with the same faecal inoculum and substrate. Fermentor 1 was shifted in the sequence pH 5.5, 6.0, 6.4, 6.9 (upshift) and fermentor 2 in the sequence pH 6.9, 6.4, 6.0, 5.5 (downshift). Samples were collected daily for short chain fatty acid analysis and DNA extraction was performed at days 0, 3, 6, 9, and 12 for qPCR and Illumina MiSeq-based sequencing of 16S rRNA gene amplicons. For each substrate, separate experiments were performed using faecal inocula from three different healthy volunteers (D1, D2 and D3)

Continuous flow anaerobic fermentors were used to investigate the impact of single polysaccharide substrates upon the species composition of gut microbiota from healthy human volunteers. Two fermentors were run in parallel in each experiment; these received an identical faecal inoculum, but one was shifted in the sequence pH 5.5, 6.0, 6.4, 6.9 (upshift) and the other in the sequence pH 6.9, 6.4, 6.0, 5.5 (downshift), with pH shifts being applied at 3-day intervals (Fig. 1). Apple pectin or inulin was provided continuously as the sole added carbohydrate energy source through the supply of fresh medium at a rate of one turnover per day (see Methods). Separate experiments were conducted for each substrate with faecal inocula from three different healthy volunteer donors, with the same three donors being used for the two sets of experiments.

### Impact of pH changes

**Fig. 2:**
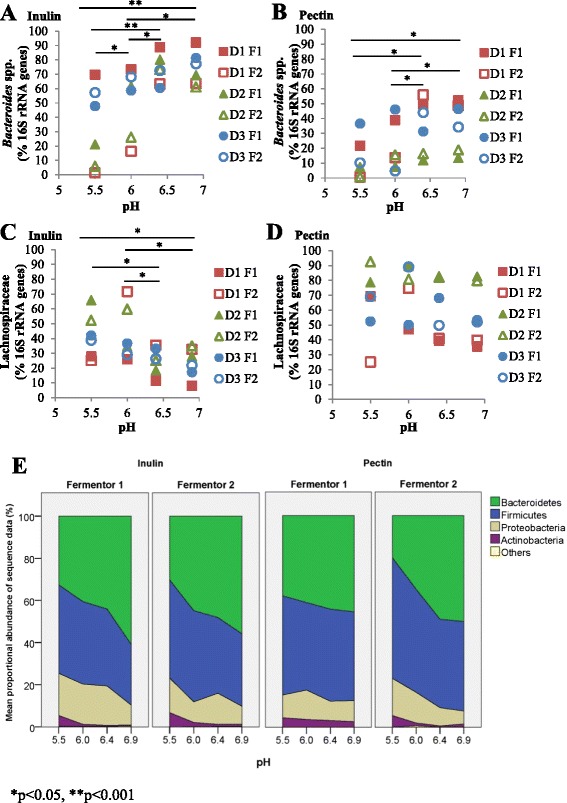
Effect of pH on microbial community composition. 16S rRNA gene-targeted qPCR data are shown for *Bacteroides* spp. (**a**) with inulin and (**b**) with apple pectin as energy sources and for Lachnospiraceae (**c**) with inulin and (**d**) with pectin. These refer to 12 fermentor runs (F1 (upshift), F2 (downshift) fermentors run in parallel for donors D1, D2 and D3 with pectin or inulin) *P <0.05, **P <0.001 (ANOVA, see Methods). Results from Illumina MiSeq sequencing of 16S rRNA gene amplicons are shown in (**e**) at the phylum level for F1 and F2 fermentors for each substrate; these revealed significant effects of pH when analyzed by ANOVA (see text). MiSeq data represent merged data from the same 12 fermentor runs, but also include four additional (repeat) runs for D2 inulin (F1, F2) and D3 pectin (F1, F2). Combined phylum and family level results from the sequence data are also shown for each substrate in Additional file 1: Figure S1. A list of operational taxonomic units obtained from analysis of 16S rRNA gene amplicon sequences for all samples is given in Additional file 2: Table S1

Changes in microbiota composition were assessed by analysis of bacterial 16S rRNA genes using qPCR and Illumina MiSeq sequencing of barcoded amplicons (see Methods). Data from pH upshift and pH downshift regimes for all donors are shown in Fig. 2 for broad bacterial groups. With inulin as a substrate there was a significant effect of pH upon the proportion of Bacteroidetes (from sequence data, ANOVA, *P* = 0.0025) and of *Bacteroides* plus *Prevotella* (as estimated by qPCR, ANOVA, *P* = 0.00037) (Fig. 2a,e). The percentage of Bacteroidetes sequences increased significantly between pH 5.5 and pH 6.9 both in F1 upshift (*P* = 0.019) and in F2 downshift (*P* = 0.031) fermentors (Fig. 2e). This is consistent with findings from previous *in vitro* studies in which starch was the major substrate supplied [11, 13]. pH had the opposite effect on the proportion of Actinobacteria sequences (ANOVA *P* = 0.0078), which decreased significantly at pH 6.9 compared with pH 5.5 both in F1 (*P* = 0.036) and F2 (*P* = 0.014) fermentors.

With pectin as a substrate, pH again had a significant effect on the percentage of Bacteroidetes sequences (ANOVA *P* = 0.0068); for this substrate, however, an increase in percentage Bacteroidetes sequences between pH 5.5 and pH 6.9 was seen only for the F2 downshift fermentors (*P* = 0.001) and not for the F1 upshift fermentors (Fig. 2e). Possible explanations for this intriguing effect of pH order are considered in the Discussion. Sequence analysis did not show a significant effect of pH upon percentage Firmicutes or Proteobacteria sequences for either substrate, although qPCR indicated a significant decrease in Lachnospiraceae between pH 5.5 and 6.9 with inulin as a substrate in F1 fermentors (Fig. 2c).

### Responses at the operational taxonomic unit (OTU) level

**Fig. 3:**
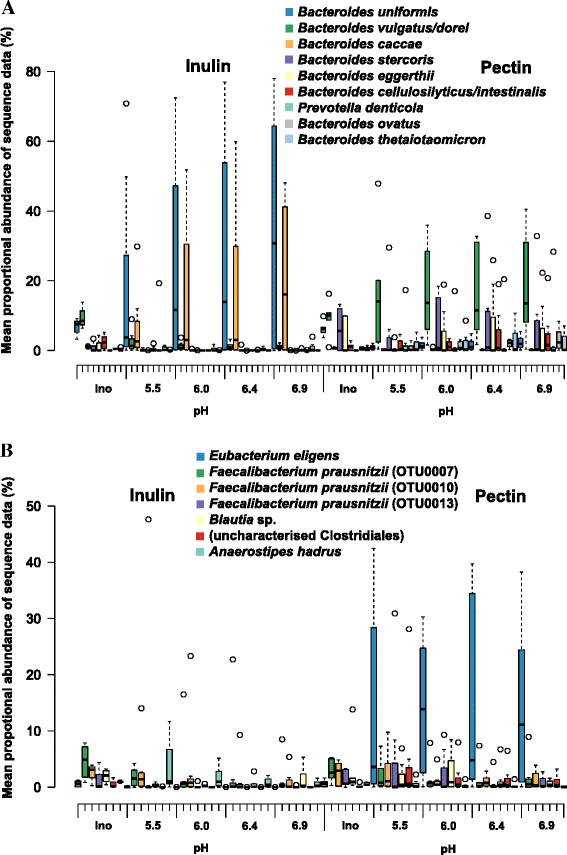
Bacteroidetes (**a**) and Firmicutes (**b**) changes across pH at the operational taxonomic unit (OTU) level. Merged data derived from Illumina MiSeq sequencing of 16S rRNA gene amplicons are shown from a total of 16 fermentor runs (F1 (upshift), F2 (downshift) fermentors run in parallel for donors D1, D2 and D3 with pectin or inulin as substrates, plus additional repeat runs for D2 inulin (F1, F2) and D2 pectin (F1, F2)). Ino = inoculum. Corresponding OTU numbers can be found in Additional file 3: Table S2 and a list of OTUs obtained from analysis of 16S rRNA gene amplicon sequences for all samples is given in Additional file 2: Table S1. Centre lines show the medians; box limits indicate the 25th and 75th percentiles as determined by R software; whiskers extend 1.5 times the interquartile range from the 25th and 75th percentiles, outliers are represented by dots

**Fig. 4:**
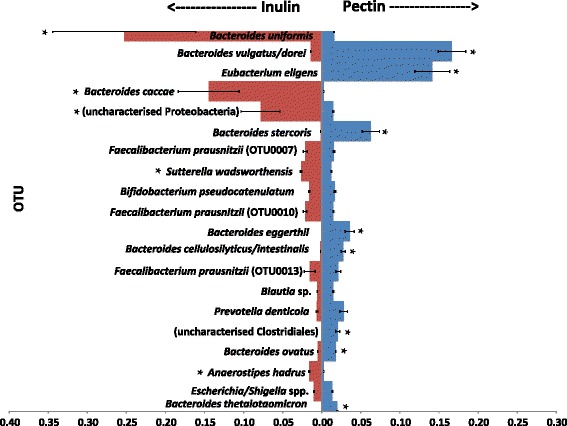
Mean proportional abundance of the top 20 most abundant operational taxonomic unit (OTUs) in inulin and pectin fermentor samples. Based on the 16 fermentor runs described in Fig. 3. Corresponding OTU numbers can be found in Additional file 3: Table S2 and the complete list of OTUs obtained from analysis of 16S rRNA gene amplicon sequences for all samples is given in Additional file 2: Table S1. Mean values and standard deviations are shown for the three donors. * Significant *P* value after Benjamini-Hochberg correction for false discovery rate (Metastats analysis)

When analyzed at the phylum or family level (Fig. 2e, Additional file 1: Figure S1), the Illumina sequence data do not suggest a major effect of the polysaccharide substrate supplied upon community composition. When viewed at the OTU level (at 97 % sequence similarity), however, a high degree of specificity is evident in the response to each substrate (Fig. 3). Careful manual curation of the OTUs allowed us to generate close approximations to species in many cases, although not all recognized species could be separated (e.g. *B. vulgatus*/*B. dorei*) while some less defined species (notably *F. prausnitzii*) were represented by several OTUs (Additional file 2: Table S1). Two OTUs derived from *Bacteroides* (related to *B. uniformis* and *B. caccae*, *P* = 0.001 and 0.001, respectively) became strongly enriched in the inulin fermentors (Fig. 4, Additional file 3: Table S2), whereas six different OTUs (*B. vulgatus/dorei*, *B. stercoris*, *B. eggerthii*, *B. cellulosilyticus*/*intestinalis*, *B. ovatus*, and *B. thetaiotaomicron*, *P* = 0.001, 0.001, 0.003, 0.001, 0.001, and 0.001, respectively) became enriched in the pectin fermentors. The impact of pH is clearly seen in the inulin fermentors for *B. uniformis* and *B. caccae* (Fig. 3a), for which the proportional representation increased with increasing pH. Within the Firmicutes, one OTU in particular, derived from *Eubacterium eligens*, and a less abundant, uncultured relative of *Roseburia* (OTU0016), were significantly enhanced by pectin relative to inulin (*P* = 0.001 for both OTUs). Firmicutes OTUs that benefited from the provision of inulin included *Anaerostipes hadrus* (OTU0018, *P* <0.002; Fig. 4, Additional file 3: Table S2, Additional file 4: Table S3). Based on the linear discriminant analysis effect size (LEfSe) method, several other Firmicutes, including one *F. prausnitzii* OTU (OTU0037, *P* <0.001), were also increased with inulin (Additional file 4: Table S3).

### Interspecies competition within individual microbiota

**Fig. 5:**
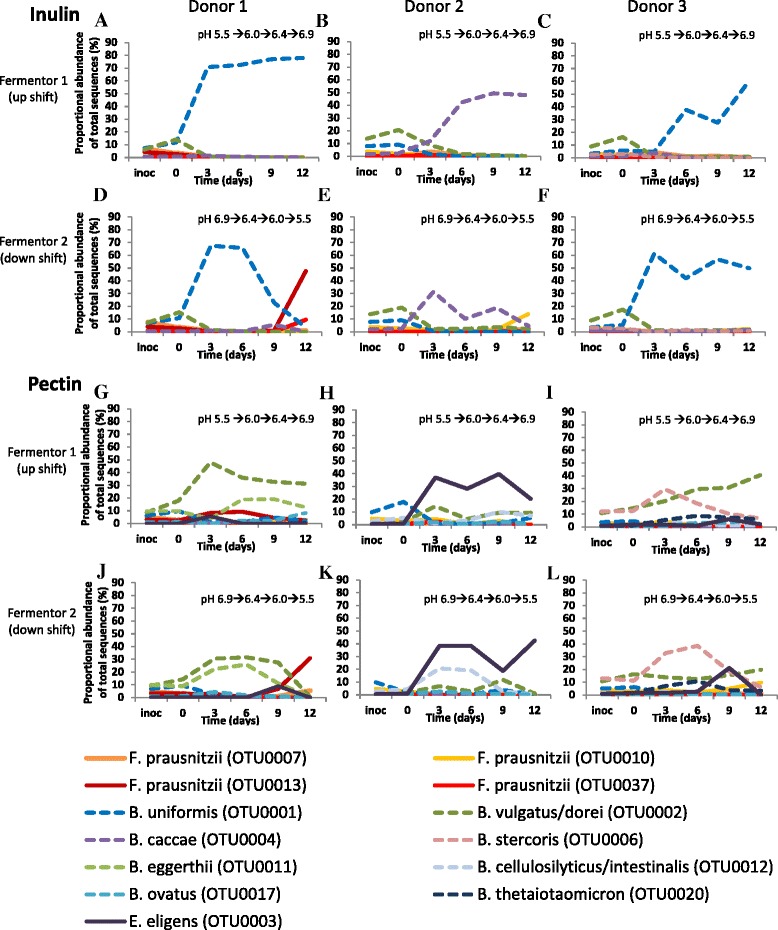
Microbial competition within individual fermentor runs. Changes in *Bacteroides* species and in the Firmicutes bacteria *F. prausnitzii* and *Eubacterium eligens* are shown for 12 fermentors (as described in Fig. 2a–d) that involved inocula from donors D1, D2 and D3. The sequence of imposed pH changes is shown for each run. These plots show the proportional abundance of OTUs determined from analysis of 16S rRNA gene amplicon sequences (Additional file 2: Table S1). Three *F. prausnitzii* OTUs and one *E. eligens* OTU were detected in inocula from all three donors, while a fourth *F. prausnitzii* OTU0013 was also detected in D1 and D3 inocula. Similarly, four *Bacteroides* OTUs (*B. uniformis*, *B. vulgatus*/*dorei*, *B. caccae* and *B. ovatus*) were present in inocula from all three donors. Of the remaining four *Bacteroides* OTUs shown, *B. cellulosilyticus*/*intestinalis* was not detected in D1 inocula, *B. thetaiotaomicron* and *B. stercoris* were not detected in D1 and D2 inocula, and *B. eggerthii* was not found in D3 inocula

Changes within individual experiments are shown in Fig. 5 for selected OTUs. These illustrate the complexity of the competitive interactions that occur between bacterial strains within these microbial communities, and the influence of variation in composition between individual microbial communities. It can be seen (Fig. 5, Additional file 2: Table S1) that the two *Bacteroides* species found to be promoted by inulin did not co-exist in the same fermentor samples, with *B. uniformis* being dominant in the D1 and D3 experiments and *B. caccae* in the D2 experiments. Interestingly, both *B. uniformis* and *B. caccae* were detected in all three inocula. This suggests that differences at the strain level are also important, e.g. the D2, but not D1 or D3, inocula harboured a *B. caccae* strain that can outcompete the *B. uniformis* strains present during growth on inulin.

The situation was more complex in the pectin-fed fermentors. *B. vulgatus*/*dorei* was apparently co-dominant with *B. eggerthii* in the D1 fermentors, with *B. cellulosilyticus*/*intestinalis* in the D2 fermentors and with *B. stercoris* in the D3 fermentors, with some indication of co-existence of co-dominant species at the same time-points. While *B. vulgatus*/*dorei* was detected in inocula from all three donors, *B. stercoris* and *B. thetaiotaomicron* were detected only in D3 and *B. eggerthii* and *B. cellulosilyticus*/*intestinalis* were not detected in D3 and D1 inocula, respectively. Thus, the emergence of different co-dominant OTUs appears to reflect the variable abundance of different *Bacteroides* species and strains in the inocula (Fig. 5, legend). *Eubacterium eligens* was able to compete with the *Bacteroides* species for pectin at certain pH values in all experiments, but was particularly successful in experiments with the D2 inoculum. In some of the downshift (F2) fermentors the dominance of the *Bacteroides* spp. was clearly curtailed or abolished at pH 5.5, and this was mirrored by a sharp increase in the abundance of *F. prausnitzii* OTUs at this pH. This phenomenon can be seen for D1_F2_Inulin, D2_F2_Inulin, and D1_F2_Pectin (Fig. 5d, e, j).

Targeted qPCR detection of selected groups (Fig. 2a–d) and species (Additional file 5: Figure S2, Additional file 6: Figure S3) further confirmed the findings obtained from amplicon sequencing. *E. eligens* was undetectable in inulin fermentors but was prominent in pectin fermentors, especially for D2, while the increase in *F. prausnitzii*, noted above, in certain fermentor communities at particular time points was again apparent (Additional file 5: Figure S2, Additional file 6: Figure S3).

### Impacts on community diversity

**Fig. 6:**
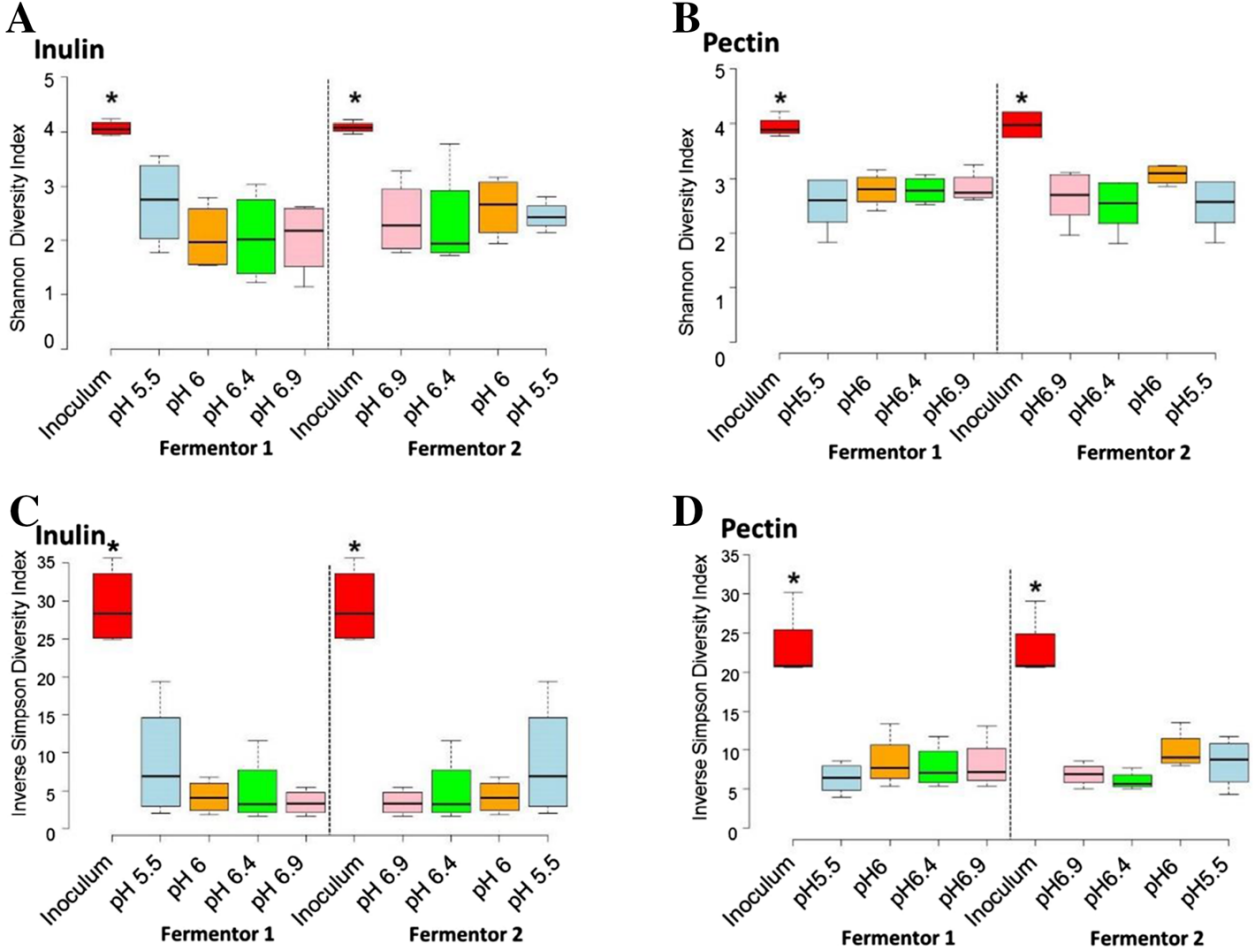
Bacterial diversity across pH range in inulin and pectin fermentors using both the Shannon index (**a**, **b**) and inverse Simpson index (**c**, **d**). Centre lines show the medians; box limits indicate the 25th and 75th percentiles as determined by R software; whiskers extend 1.5 times the interquartile range from the 25th and 75th percentiles, outliers are represented by dots. * Indicates significant difference between the faecal inocula and all fermentor pH conditions (ANOVA, *P* <0.001). Diversity estimates were derived from 16S rRNA gene OTU analysis of the 16 fermentor runs described in Fig. 3 and shown in Additional file 2: Table S1

The diversity of the fermentor community was reduced by comparison with the faecal inoculum in all experiments (*P* <0.001), but remained stable over time between days 3 and 12 (Shannon indices of between 2 and 3 compared with around 4 for the inoculum). Diversity was apparently unaffected by the imposed pH shifts, but Shannon diversity indices were significantly higher for pectin-fed than inulin-fed fermentors (*P* = 0.001; Fig. 6).

### Short chain fatty acids (SCFA)

**Fig. 7:**
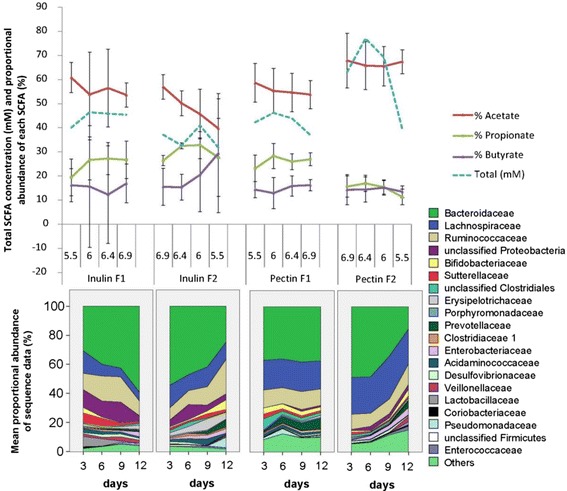
Short chain fatty acids (SCFA) in upshift and downshift fermentors. Mean SCFA values (means and standard deviations) and proportional abundance of bacterial families based on sequence analysis of 16S rRNA gene amplicons are shown for the 16 fermentor runs described in Fig. 3. Significant changes in % SCFA (from ANOVA) are discussed in the text. ANOVA revealed significant decreases in % Bacteroidaceae between pH 6.9 and pH 5.5 in inulin fermentors F1 (*P* = 0.015) and F2 (*P* = 0.012), but with pectin only for the F2 (downshift) fermentors (*P* = 0.0001). % Bifidobacteriaceae and % Lachnospiraceae increased significantly at pH 5.5 compared with pH 6.9 in F2 inulin (*P* = 0.007) and F1 inulin (*P* = 0.025) fermentors, respectively

SCFA concentrations were measured for all time points and the mean values across the whole pH range shown in Additional file 7: Table S4. This revealed that the proportion of *Bacteroides* spp. and *Prevotella* spp. present in the fermentor community (as determined by qPCR) was positively correlated with the concentration of propionate measured at the same time point (R^2^ = 0.558; Additional file 8: Figure S4A). The butyrate concentration was only weakly correlated to the combined abundance of two prominent groups of butyrate-producing bacteria, *F. prausnitzii* and *Roseburia* spp. (R^2^ = 0.278), which may reflect the known contribution of other Firmicutes to butyrate production [15] (Additional file 8: Figure S4B). SCFA production from pectin at low pH was apparently unaffected by the direction of the pH change, but altered SCFA proportions were observed with inulin as a substrate at pH 5.5 after 12 days in the downshift fermentors (with a significant decrease in the proportion of acetate, *P* = 0.02; Fig. 7). Family level changes derived from the MiSeq data were in line with the shifts in microbiota composition reported earlier in Fig. 2 (Fig. 7, legend).

## Discussion

Several different approaches can be employed to understand and predict the selective influence of ND carbohydrates upon the gut microbiota. Analysis of carbohydrate active enzyme (CAZyme, Carbohydrate Active Enzymes database [16, 17], URL http://www.cazy.org/) complements from individual genomes [18] and growth tests on isolated species [19] may be indicative, but cannot predict how different organisms will compete and interact within the complex intestinal community. At the other extreme, *in vivo* feeding experiments cannot readily distinguish effects mediated via the gut environment, for example, changes in gut transit or pH, from the direct selective effects of the substrate. Furthermore, *in vivo* nutritional studies do not provide precise control over the substrates available to the microbiota, since endogenous substrates such as mucin and other food components will always be present. The approach that we have adopted here of using *in vitro* continuous flow fermentors to study the human intestinal microbiota as a microcosm has the considerable advantage of allowing precise control over both substrate supply and pH.

Detailed investigations into polysaccharide utilization have so far been mainly undertaken for *Bacteroides* spp. among predominant human gut anaerobes [20], although more recently studies have also been conducted in the Firmicutes representative *Eubacterium rectale* [21]. In *Bacteroides* spp., genes responsible for the degradation of particular polysaccharides are organized at polysaccharide utilization loci (PULs) that also encode transport functions and transcriptional regulators. Individual *Bacteroides* genomes typically possess large numbers of PULs (over 80) that are concerned with degradation of different host- and diet-derived polysaccharides [22, 23]. Variations in PUL distribution between species have been demonstrated to correlate with the ability to utilize particular polysaccharides *in vitro* and in gnotobiotic animal models [23–25]. Our results show that all of the eight most abundant *Bacteroides* OTUs present in the initial faecal microbiota gave a strong differential response in the mixed fermentor communities, being stimulated either by inulin (in two cases) or by pectin (in six cases). Similar selectivity was evident for many Firmicutes. Among the top 38 OTUs (each representing 0.5 % or more of the total sequences), five Firmicutes OTUs increased in abundance with inulin, while a further five Firmicutes OTUs increased with pectin (Additional files 3 and 4: Tables S2 and S3).

Previous studies on cultured isolates indicated that pectin utilization is widespread among *Bacteroides* spp., but relatively uncommon among human colonic Firmicutes [26, 27]. Nevertheless, the strong and highly specific enrichment of the pectin-utilizing Firmicutes species *E. eligens* [26, 27] could provide the basis for an effective prebiotic strategy while enrichment of one *F. prausnitzii* OTU by pectin was also observed here. Inulin stimulated the Firmicutes species *F. prausnitzii* and *A. hadrus* (strains related to the isolate SS2/1) as has also been reported in an *in vivo* human study [10, 15]. Both species are butyrate-producing bacteria that offer potential benefits to health, via anti-inflammatory action in the case of *F. prausnitzii* [28], and via conversion of D-lactate to butyrate in the case of *A. hadrus* [29, 30]. In addition to promoting particular Bacteroidetes and Firmicutes OTUs, inulin also promoted some Proteobacteria, including *Sutterella wadsworthensis*. This may warrant further investigation as *S. wadsworthensis* has been isolated not only from healthy individuals but also from gastrointestinal disease states [31].

Many studies have shown that inulin has a bifidogenic effect *in vivo* [9, 32–34] and there is also a report suggesting that pectin can be bifidogenic [35]. Interestingly, we found no evidence in these experiments for an overall stimulation of bifidobacteria by inulin or pectin either from sequence analysis, even though *B. pseudocatenulatum* was detected as one of the 10 most abundant OTUs, or from qPCR monitoring of the *Bifidobacterium* genus. Since the representation of bifidobacteria was greatest at pH 5.5 in the inulin fermentors (Fig. 7), it therefore seems likely that the promotion of bifidobacteria by inulin that is observed *in vivo* depends critically on acidification in the colonic lumen that results from SCFA production [12]. At pH values closer to neutrality our evidence suggests that other bacteria will tend to out-compete bifidobacteria for inulin. It is also known that the chain length of inulin is critical in determining its utilization by isolated gut anaerobes [19], which can complicate the comparison of different studies. Many isolated bifidobacteria cannot utilize long chain inulin, although they are able to benefit from fructo-oligosaccharide breakdown products via cross-feeding in co-cultures [36]. In general, metabolic cross-feeding involving partial degradation products, fermentation products and growth factors, but also inhibitory interactions, can be expected to have played a role in the community changes occurring in these studies [37, 38].

The overall decrease in microbiota diversity in the fermentor community relative to the faecal inoculum (seen from Fig. 6) is assumed to result mainly from the supply of a single substrate as energy source. By contrast, typical human diets supply a very wide variety of types of fibre and a wide array of host-derived glycans and proteins (especially mucins). Another important factor is likely to be the relative stability of conditions within the fermentor compared with the fluctuations that occur *in vivo* as a result of periodic meals.

Our experiments also show that pH exerts a strong influence on competition between bacteria from different phyla or families that share the ability to utilize the same polysaccharide. This effect was particularly clear for the inulin fermentors, where the proportion of Bacteroidetes 16S rRNA gene copies fell from around 60 % at pH 6.9 to around 30 % at pH 5.5. This suppression of *Bacteroides* spp. at slightly acidic pH appears to reflect growth inhibition by SCFAs at pH values below 6 [11, 13]. Interestingly, this trend was not evident for the pectin upshift fermentors where *E. eligens* was apparently able to compete with pectin-utilizing *Bacteroides* spp. across the pH range. The correlation between *Bacteroides* representation in the community and the proportion of propionate that was seen here has also been noted for faecal samples from an *in vivo* study [6]. This is assumed to reflect the dominant role of the succinate pathway, which is found mainly in the Bacteroidetes among human colonic bacteria, in the formation of propionate from carbohydrates [39]. Thus, there was considerable functional redundancy with respect to propionate formation, as substrate-driven changes in the dominant species of Bacteroidetes appeared to have little influence. On the other hand, the species level changes seen here could potentially have many other effects on the host, including those via immune signalling and metabolite transformation and which will warrant investigation in future studies.

We observed some asymmetry in the response of the microbial community to low pH in parallel downshift and upshift fermentors that received the same inoculum. A possible explanation for this lies in the initial reduction in community diversity in the fermentors, discussed above. Theoretical modelling suggests that diversity will also have decreased at the strain level as strains that have optimal characteristics for pH tolerance and substrate utilization are selected [40]. Thus, the community at the start of the pH 5.5 phase in the downshift fermentors was considerably less diverse than the inoculum that initiated the pH 5.5 phase (*P* <0.005) in the upshift fermentors. This lower diversity may have led to the absence of *Bacteroides* strains tolerant of pH 5.5 at the end of the downshift runs, thus allowing the growth of more low-pH tolerant competitors such as *F. prausnitzii* [27, 40] and perhaps explaining the observed ‘blooms’ in *F. prausnitzii* that were seen in several fermentor runs (Fig. 5). Interestingly, sudden shifts in the ratio of *Bacteroides* to *F. prausnitzii* have also been reported *in vivo* in human subjects [41].

In summary, we have shown that two ND carbohydrates, inulin and pectin, promote very different community profiles when supplied as sole energy sources to human colonic microbial communities under conditions of controlled pH and turnover. Notably, these differences were found to lie at the species level rather than at the phylum or family level. We found very little overlap between the species stimulated by the two substrates, implying that evolution in the highly competitive environment of the large intestinal microbiota has favoured a high degree of nutritional niche specialization at the species level. At the same time, it is evident that a number of phylogenetically distant organisms belonging to different phyla have evolved convergently to gain the ability to utilize the same substrates. With a simple homopolymer (inulin) as a substrate, a single species was found to dominate the community at a given pH, but several species were apparently able to co-exist on pectin at the same pH. It is likely that the extreme chemical complexity of pectin [42] creates multiple nutritional niches which can explain the greater microbiota diversity seen in the pectin-fed relative to the inulin-fed communities. Our experiments also show that the species that become dominant with a given substrate can vary for individual microbial communities, and are likely to depend on the precise mix of competing strains within each microbiota.

## Conclusions

In view of these and other recent findings, dietary manipulation through supplementation with ND carbohydrates has considerable potential for modifying the composition of the human colonic microbiota, with the aim of benefiting health. However, our findings show that it is crucial to define the profile of bacterial species promoted by an intended prebiotic, as even closely related species within the human colonic microbiota have evolved distinct substrate preferences*.* Bacterial populations that become promoted are likely to include species from outside the group originally targeted, and may potentially include harmful as well as beneficial organisms and also species whose effects on the host are unknown. Our work also provides a compelling rationale for pursuing ‘synbiotic’ approaches, whereby prebiotic substrates are specifically matched with beneficial microbes that are capable of utilizing them in order to enhance their colonization and activities *in vivo*. This could involve specific combinations of prebiotics and probiotics, or target populations of resident bacteria. First, however, it is necessary to identify which species within the complex gut microbiota are likely to respond to a given dietary manipulation. The approaches described here can thus help us to focus the necessary research into the health consequences of dietary manipulation to the most relevant bacterial species.

## Methods

### Simulated human colonic fermentor studies

Two single-stage fermentor systems, with a working volume of 250 mL, were set-up as described previously using a medium based on that of Macfarlane et al. [43], containing 0.6 % (w/v) peptide [13]. In the present experiments, however, the only added carbohydrate energy source was either apple pectin (Sigma) or inulin (Oliggo-Fiber DS2, avDP <10, Cargill Inc.) at 0.5 % (w/v). The fermentor culture vessels were maintained under a stream of CO_2_ at a constant temperature of 37 °C using a thermal jacket. Both the medium reservoir and fermentor culture vessel were mixed by internal stirrer bars powered by external stirring units. The volume of the culture was kept constant at 250 mL with a constant flow of fresh medium at a turnover of 250 mL/day. A SCFA mix was added initially in the apple pectin fermentors, containing 33 mM acetate, 9 mM propionate and 1 mM each of *iso*-butyrate, *iso*-valerate and valerate. The pH of the fermentor vessels were monitored and controlled using a pH controller that delivers either 0.1 M HCl or 0.1 M NaOH solutions to maintain the pH at 5.5 ± 0.2, 6.0 ± 0.2, 6.4 ± 0.2 and 6.9 ± 0.2 for a period of 3 days at each pH. One fermentor vessel (fermentor 1) started from the low pH (5.5) at day 0 with pH increasing over time (upshift) (Fig. 1). The other (fermentor 2) started from the highest pH (6.9) at day 0 with pH decreasing over time (downshift). Inocula were from fresh faecal samples and were prepared by mixing 5 g of faeces in 10 mL of 50 mM phosphate buffer (pH 6.5) under O_2_ free CO_2_ containing 0.05 % cysteine, using gentle MACS™ M Tubes (MACS Miltenyl Biotec). Faecal samples were donated by three healthy adult volunteers who had no history of colonic disease and had consumed no drugs known to influence the microbiota for at last 3 months prior to the sampling date. All volunteers were omnivores, one male (64 years old) and two female (53 and 42 years old).

### DNA extraction

Each fermentor sample collected was immediately processed using the FastDNA Spin kit (MP Biomedicals). The samples (460 μL) were placed in lysing matrix E tubes and 978 μL of sodium phosphate buffer and 122 μL MT buffer were added to each tube, which was processed following the manufacturer’s instructions. The DNA was eluted in 50 μL FastPrep elution buffer. Faecal DNA was quantified by Nanodrop mass spectrophotometry.

### PCR amplification and Illumina MiSeq sequencing

The extracted DNA was used as a template for PCR amplification of the V1-V2 region of bacterial 16S rRNA genes using the barcoded fusion primers MiSeq-27F (5′-AATGATACGGCGACCACCGAGATCTACACTATGGTAATTCCAGMGTTYGATYMTGGCTCAG-3′) and MiSeq-338R (5′-CAAGCAGAAGACGGCATACGAGAT-barcode-AGTCAGTCAGAAGCTGCCTCCCGTAGGAGT-3′), which also contain adaptors for downstream Illumina MiSeq sequencing. Each of the samples was amplified with a unique (12 base) barcoded reverse primer.

Initial PCR amplification was undertaken with New England BioLabs Q5 High-fidelity DNA Polymerase, utilizing a per-reaction mix of DNA template (1 μL), 5X Q5 Buffer (5 μL), 10 mM dNTPs (0.5 μL), 10 μM F Primer (1.25 μL), 10 μM R Primer (1.25 μL), Q5 Taq (0.25 μL) and sterile, deionised water (15.75 μL) to a final volume of 20 μL. PCR cycling conditions were as follows: 2 minutes at 98 °C; 20 cycles of 30 s at 98 °C, 30 s at 50 °C, 120 s at 72 °C; end with 5 mins at 72 °C then holding temperature at 10 °C. Quadruplicate PCR reactions per DNA sample were set up. Following confirmation of adequate and appropriately sized products, the quadruplicate reactions were pooled and ethanol precipitated. The pooled amplicons were then quantified using a Qubit 2.0 Fluorometer (Life Technologies Ltd) and a sequencing mastermix was created using equimolar concentrations of DNA from each sample. Sequencing was carried out on an Illumina MiSeq machine, using 2 × 250 bp read length, at the Wellcome Trust Sanger Institute (Cambridgeshire, UK). Sequence data has been deposited in the European Nucleotide Archive and is available under study accession number ERP010892, and sample accession numbers ERS782738 to ERS782828 (Additional file 2: Table S1).

The data obtained from Illumina MiSeq sequencing was analyzed using the mothur software package [44] and their MiSeq SOP [45]. Forward and reverse reads generated by sequencing were assembled into paired read contigs. Resulting contigs that were shorter than 270 bp, longer than 480 bp, contained ambiguous bases, or contained homopolymeric stretches of longer than 7 bases, were all removed. Unique sequences were then grouped together and aligned against the SILVA reference database. Pre-clustering (diffs = 3) was carried out to reduce the impact of sequencing errors and the OTUs were generated at a 97 % similarity cut-off level. Taxonomic classifications for each OTU from phylum to genus level were assigned using the RDP reference database (release 10) [46]. To assign species-level classifications, representative sequences for each OTU were generated using the “get.oturep” command in mothur and then searched against the NCBI Nucleotide database using MegaBLAST. Species-level classifications were assigned to OTUs where there was a greater than 99 % similarity to a reference sequence derived from a cultured isolate, and no similarity at greater than 97 % to any other cultured species in order to allow for the 3 % variation within each OTU. For OTUs where there were more than one species within a 3 % similarity margin “spp.” or multiple species names were used to indicate that we could be capturing more than one characterized species within a given OTU. Chimeric molecules created during PCR amplification as well as reads from chloroplast, mitochondria, archaea, eukaryote and unknown sequences were removed from the dataset [47]. The resulting dataset had a total of 1,721,766 sequences with a range of 8767–27679 sequences per sample. All samples were rarefied to 8767 to ensure equal sequencing depth for all comparisons. The final OTU-level results are shown in Additional file 2: Table S1. Metastats analysis [48] was used to determine any OTUs that were significantly different between two sample cohorts, and *P* values were corrected with the Benjamini-Hochberg method [49] to allow for the false discovery rate over multiple comparisons. In addition, significant differences across all cohorts were identified using LEfSe analysis [50]. The Shannon diversity index and inverse Simpson diversity index were used to calculate bacterial diversity per sample.

### Quantitative real-time PCR (qPCR)

qPCR was performed in duplicate with iTaq^TM^ Universal SYBR^®^ Green Supermix (Bio-Rad) in a total volume of 10 μL with primers at 500 nM and 5 ng of DNA in optical-grade 384-well plates sealed with optical sealing tape. Amplification was performed with a CFX384^TM^ Real-time System (Bio-Rad) with the following protocol: one cycle of 95 °C for 3 min, 40 cycles of 95 °C for 5 s and annealing temperature as per Additional file 9: Table S5 for 30 s, 1 cycle of 95 °C for 10 s, and a stepwise increase of the temperature from 65 °C to 95 °C (at 5 s per 0.5 °C) to obtain melt curve data. As described previously [10], standard curves consisted of 10-fold dilution series of amplified bacterial 16S rRNA genes from reference strains. Samples were amplified with universal primers against total bacteria and specific primers against *Bifidobacterium* spp., *Bacteroides* spp., *Prevotella* spp., Clostridial cluster XIVa spp. (Lachnospiraceae), *F. prausnitzii*, *A. hadrus*, *E. eligens* and *E. rectale*/*Roseburia* spp. (Additional file 9: Table S5). The abundance of 16S rRNA genes was determined from standard curves and specific bacterial groups were expressed as a percentage of total bacteria as determined by the universal primers. The detection limit was determined with negative controls containing only herring sperm DNA. qPCR were performed in duplicates and the data were analysed using BioRad CFX manager software.

### Short chain fatty acid (SCFA) analysis

SCFA formation was assessed in fermentor samples by gas chromatography as described previously [51]. Briefly, following derivatisation of the samples using N-tert-butyldimethylsilyl-N-methyltrifluoroacetamide, the samples were analysed using a Hewlett Packard gas chromatograph fitted with a fused silica capillary column with helium as the carrier gas.

### Statistical analyses

SCFA, MiSeq and qPCR data from these experiments were analysed by ANOVA with donor, fermentor, and donor and time within fermentor as random effects, and with pH, run (upshift or downshift), and their interaction as fixed effects. When an effect was significant (*P* <0.05) mean values were then compared by post-hoc *t*-test based on the output from the ANOVA analysis.

## Additional files

Additional file 1: Figure S1. Effect of pH on microbial community composition at the (A) phylum and (B) family level. (DOCX 240 kb) Additional file 2: Table S1. Proportional abundance of each operational taxonomic unit (OTU; in %) per sample (97 % OTU cut-off). (XLSX 1538 kb) Additional file 3: Table S2. Metastats analysis of the top 38 most abundant operational taxonomic unit (OTUs; those accounting for more than 0.5 % of total proportional abundance) between pectin and inulin fermentors. (DOCX 41 kb) Additional file 4: Table S3. Linear discriminant analysis effect size of different sample cohorts – faecal inoculum, pectin and inulin. (DOCX 40 kb) Additional file 5: Figure S2. Changes in the human faecal microbial community composition with apple pectin as monitored by qPCR. (DOCX 938 kb) Additional file 6: Figure S3. Changes in the human faecal microbial community with inulin monitored by qPCR. (DOCX 932 kb) Additional file 7: Table S4. Summary table of mean short chain fatty acid (SCFA) concentrations (mM) in pectin and inulin fermentors across all pH values. (DOCX 38 kb) Additional file 8: Figure S4. Correlation of metabolite concentrations with key propionate and butyrate producers as determined by qPCR in both apple pectin and inulin fermentors. (DOCX 43 kb) Additional file 9: Table S5. List of qPCR primers used in this study including information on primer target, sequence, annealing temperature and references. (DOCX 39 kb)
