# Supplementary material for: Modulation of the human gut microbiota by dietary fibres occurs at the species level
Source: BMC Biol. 2016 Jan 11;14:3. doi: 10.1186/s12915-015-0224-3 (PMC4709873; doi:10.1186/s12915-015-0224-3)
Supplement: Additional file 1: Figure S1. — Effect of pH on microbial community composition at the (A) phylum and (B) family level. (DOCX 240 kb) [file 12915_2015_224_MOESM1_ESM.docx]

**A. B.**


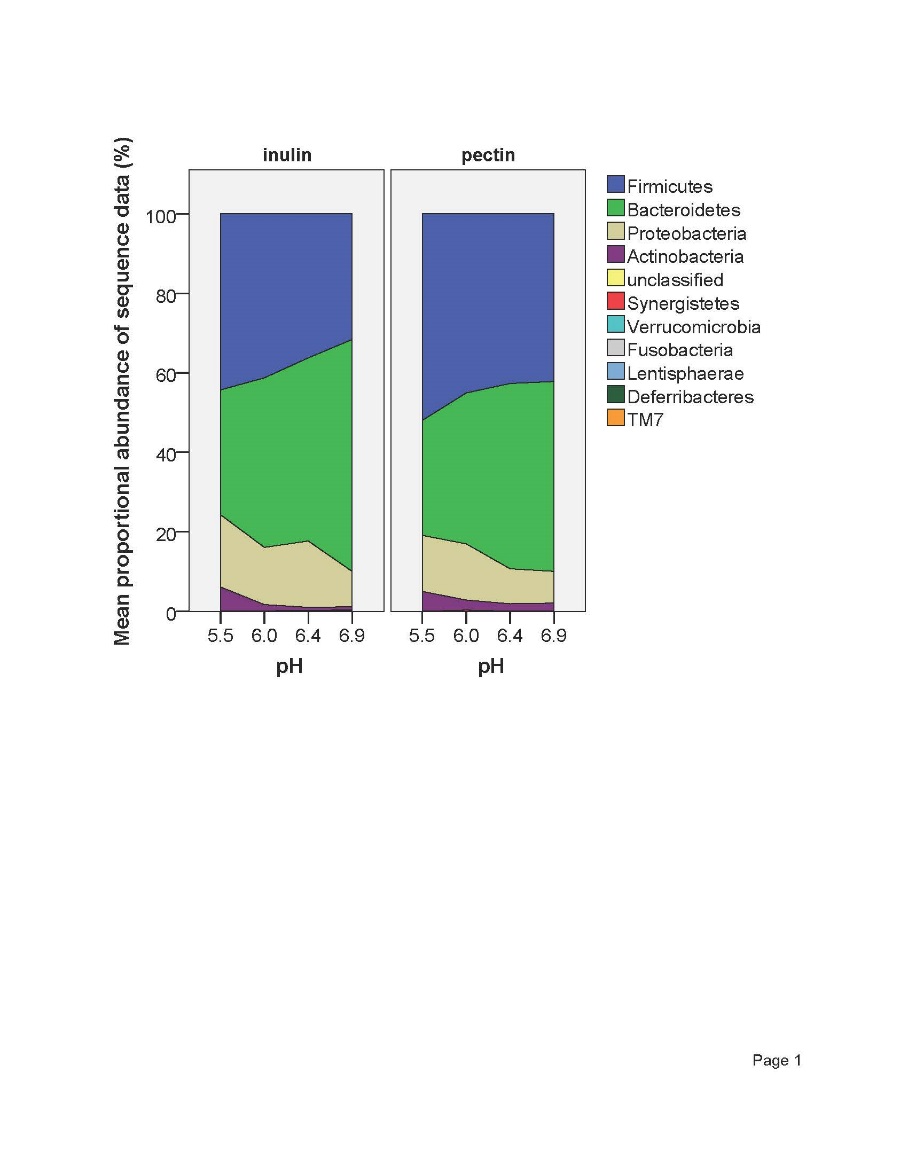

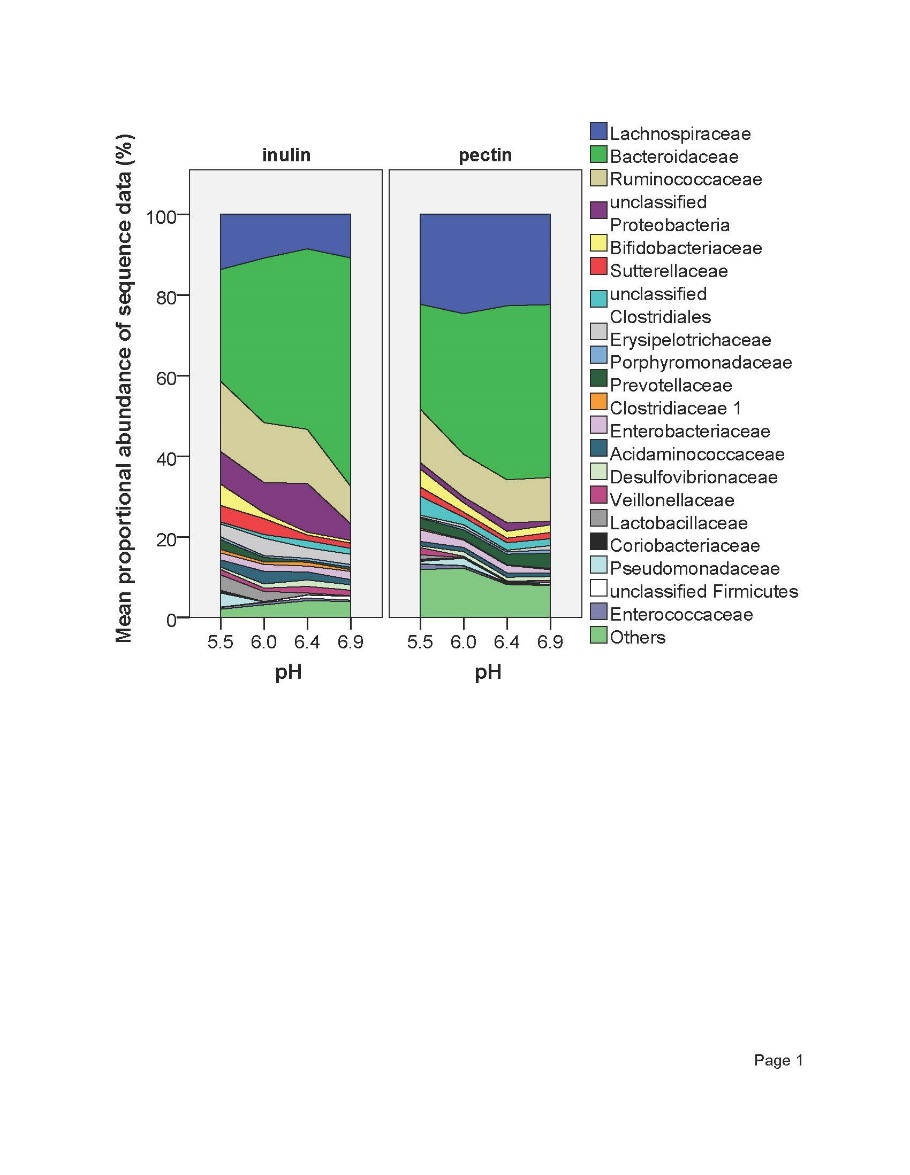


**Figure 1S. Effect of pH on microbial community composition at (A) phylum level (B) family level.**

ANOVA revealed a significant overall effect of pH (between 5.5 and 6.9) at the phylum level (A) in the inulin fermentors on the % Bacteroidetes (p= 0.025) and % Actinobacteria (p= 0.0078), and in the pectin fermentors for % Bacteroidetes (p= 0.0068). In some cases parallel pH upshift (F1) and downshift (F2) vessels gave different outcomes and the data are therefore shown separately in the main paper (Figure 2). At the family level (B) in the inulin fermentors pH (between 5.5 and 6.9) had a significant overall effect on % Bacteroidaceae (p= 0.012), % Lachnospiraceae (p= 0.042) and % Bifidobacteriaceae (p= 0.009) and in the pectin fermentors for % Bacteroidaceae (p= 0.0049).
