# Supplementary material for: Modulation of the human gut microbiota by dietary fibres occurs at the species level
Source: BMC Biol. 2016 Jan 11;14:3. doi: 10.1186/s12915-015-0224-3 (PMC4709873; doi:10.1186/s12915-015-0224-3)
Supplement: Additional file 3: Table S2. — Metastats analysis of the top 38 most abundant operational taxonomic unit (OTUs; those accounting for more than 0.5 % of total proportional abundance) between pectin and inulin fermentors. (DOCX 41 kb) [file 12915_2015_224_MOESM3_ESM.docx]

**Table 2S. Metastats analysis of the top 38 most abundant OTUs (those accounting for more than 0.5% of total proportional abundance) between pectin and inulin fermentors.** P values were corrected using the Benjamini-Hochberg method [1] to account for multiple comparisons. (OTUs number 21, 23, 34 and 39 were removed as these were considered to be contaminants derived from kit reagents [2]).

| OTU | **MegaBLAST Closest Match (Representative Seq.)** | mean(pectin) | mean(inulin) | p-value | Significant after applying Benjamini-Hochberg correction |
| --- | --- | --- | --- | --- | --- |
| Otu0001 | *Bacteroides uniformis* | 0.015702 | 0.252702 | 0.000999 | Yes |
| Otu0002 | *Bacteroides vulgatus/dorei* | 0.166548 | 0.014222 | 0.000999 | Yes |
| Otu0003 | *Eubacterium eligens* | 0.14134 | 0.000267 | 0.000999 | Yes |
| Otu0004 | *Bacteroides caccae* | 0.002381 | 0.144494 | 0.000999 | Yes |
| Otu0005 | (uncharacterised Proteobacteria) | 0.014668 | 0.078405 | 0.018981 | Yes |
| Otu0006 | *Bacteroides stercoris* | 0.062625 | 0.001194 | 0.000999 | Yes |
| Otu0007 | *Faecalibacterium prausnitzii* | 0.015591 | 0.021195 | 0.597403 | No |
| Otu0008 | *Sutterella wadsworthensis* | 0.012454 | 0.026531 | 0.022977 | Yes |
| Otu0009 | *Bifidobacterium pseudocatenulatum* | 0.016778 | 0.016218 | 0.943057 | No |
| Otu0010 | *Faecalibacterium prausnitzii* | 0.014853 | 0.021423 | 0.508492 | No |
| Otu0011 | *Bacteroides eggerthii* | 0.035727 | 0.000075 | 0.002997 | Yes |
| Otu0012 | *Bacteroides cellulosilyticus/intestinalis* | 0.027393 | 0.001693 | 0.000999 | Yes |
| Otu0013 | *Faecalibacterium prausnitzii* | 0.021009 | 0.015452 | 0.738262 | No |
| Otu0014 | *Blautia* sp. | 0.01455 | 0.005953 | 0.073926 | No |
| Otu0015 | *Prevotella denticola* | 0.027757 | 0.006167 | 0.078921 | No |
| Otu0016 | (uncharacterised Clostridiales) | 0.02056 | 0.000068 | 0.000999 | Yes |
| Otu0017 | *Bacteroides ovatus* | 0.017826 | 0.005297 | 0.000999 | Yes |
| Otu0018 | *Anaerostipes hadrus* | 0.002599 | 0.01604 | 0.001998 | Yes |
| Otu0019 | *Escherichia/Shigella* spp. | 0.013495 | 0.009949 | 0.414585 | No |
| Otu0020 | *Bacteroides thetaiotaomicron* | 0.019605 | 0.000271 | 0.000999 | Yes |
| Otu0022 | *Streptococcus* spp. | 0.010237 | 0.005461 | 0.173826 | No |
| Otu0024 | *Clostridium saudimassiliensis* | 0.000474 | 0.002356 | 0.038961 | No |
| Otu0025 | *Desulfovibrio piger* | 0.008583 | 0.010622 | 0.607393 | No |
| Otu0026 | (uncharacterised Ruminococcaceae) | 0.005507 | 0.010654 | 0.070929 | No |
| Otu0027 | *Intestinibacter bartlettii* | 0.012686 | 0.001878 | 0.002997 | Yes |
| Otu0028 | (uncharacterised Lachnospiraceae) | 0.001782 | 0.017131 | 0.006993 | Yes |
| Otu0029 | (uncharacterised Ruminococcaceae) | 0.002107 | 0.000296 | 0.000999 | Yes |
| Otu0030 | (uncharacterised Ruminococcaceae) | 0.000007 | 0.017516 | 0.000999 | Yes |
| Otu0031 | *Guyana massiliensis* | 0.000542 | 0.005279 | 0.000999 | Yes |
| Otu0032 | *Ruminococcus bromii* (93% similarity) | 0.002345 | 0.000096 | 0.001998 | Yes |
| Otu0033 | *Clostridium ramosum* | 0.001686 | 0.014639 | 0.000999 | Yes |
| Otu0035 | *Barnesiella intestinihominis* | 0.001347 | 0.002292 | 0.114885 | No |
| Otu0036 | *Lactobacillus* spp. | 0.000007 | 0.015659 | 0.100899 | No |
| Otu0037 | *Faecalibacterium prausnitzii* | 0.002388 | 0.008854 | 0.123876 | No |
| Otu0038 | (uncharacterised Ruminococcaceae) | 0.005974 | 0.00113 | 0.064935 | No |
| Otu0040 | *Acidaminococcus intestini* | 0.007496 | 0.005322 | 0.648352 | No |
| Otu0041 | *Eubacterium rectale* | 0.000506 | 0.005714 | 0.063936 | No |

1. Benjamini Y, Hochberg Y. Controlling the False Discovery Rate: A Practical and Powerful Approach to Multiple Testing. 1995;57:1:289-300.

2. Salter SJ, Cox MJ, Turek EM, Calus ST, Cookson WO, Moffatt MF, et al. Reagent and laboratory contamination can critically impact sequence-based microbiome analyses. BMC Biol. 2014;12:1.
