# Supplementary material for: Modulation of the human gut microbiota by dietary fibres occurs at the species level
Source: BMC Biol. 2016 Jan 11;14:3. doi: 10.1186/s12915-015-0224-3 (PMC4709873; doi:10.1186/s12915-015-0224-3)
Supplement: Additional file 4: Table S3. — Linear discriminant analysis effect size of different sample cohorts – faecal inoculum, pectin and inulin. (DOCX 40 kb) [file 12915_2015_224_MOESM4_ESM.docx]

**Table 3S**. **LEfSe analysis of different sample cohorts – faecal inoculum, pectin and inulin.** Cohorts indicated in the class column shows significant increase in proportional abundance of a particular OTU in comparison to the other cohorts. Average proportional abundance of each OTU (%) in the faecal inocula is also indicated. (OTUs number 21, 23, 34 and 39 were removed as these were considered to be contaminants derived from kit reagents [2]).

| OTU | Average Inoculum composition | Class | pValue | **MegaBLAST Closest Match (Representative Seq.)** |
| --- | --- | --- | --- | --- |
| Otu0001 | 6.86 | - |  | *Bacteroides uniformis* |
| Otu0002 | 8.30 | Pectin | 2.78E-10 | *Bacteroides vulgatus/dorei* |
| Otu0003 | 0.54 | Pectin | 3.45E-13 | *Eubacterium eligens* |
| Otu0004 | 0.92 | Inulin | 9.66E-08 | *Bacteroides caccae* |
| Otu0005 | 2.21 | - |  | (uncharacterised Proteobacteria) |
| Otu0006 | 2.24 | - |  | *Bacteroides stercoris* |
| Otu0007 | 3.74 | Inoculum | 0.000399 | *Faecalibacterium prausnitzii* |
| Otu0008 | 2.24 | Inulin | 0.003647 | *Sutterella wadsworthensis* |
| Otu0009 | 2.60 | - |  | *Bifidobacterium pseudocatenulatum* |
| Otu0010 | 2.73 | Inoculum | 0.016165 | *Faecalibacterium prausnitzii* |
| Otu0011 | 2.04 | - |  | *Bacteroides eggerthii* |
| Otu0012 | 1.93 | Pectin | 0.00069 | *Bacteroides cellulosilyticus/intestinalis* |
| Otu0013 | 1.20 | Pectin | 0.001284 | *Faecalibacterium prausnitzii* |
| Otu0014 | 3.39 | - |  | *Blautia* sp. |
| Otu0015 | 0.00 | - |  | *Prevotella denticola* |
| Otu0016 | 0.43 | Pectin | 9.96E-08 | (uncharacterised Clostridiales) |
| Otu0017 | 0.51 | Pectin | 0.000919 | *Bacteroides ovatus* |
| Otu0018 | 0.80 | Inulin | 2.08E-05 | *Anaerostipes hadrus* |
| Otu0019 | 0.04 | - |  | *Escherichia/Shigella* spp. |
| Otu0020 | 0.24 | - |  | *Bacteroides thetaiotaomicron* |
| Otu0022 | 0.74 | - |  | *Streptococcus* spp. |
| Otu0024 | 0.29 | - | 2.37E-06 | *Clostridium saudimassiliensis* |
| Otu0025 | 0.17 | - |  | *Desulfovibrio piger* |
| Otu0026 | 0.58 | - |  | (uncharacterised Ruminococcaceae) |
| Otu0027 | 0.66 | - |  | *Intestinibacter bartlettii* |
| Otu0028 | 0.00 | - |  | (uncharacterised Lachnospiraceae) |
| Otu0029 | 2.23 | Inoculum | 5.34E-05 | (uncharacterised Ruminococcaceae) |
| Otu0030 | 0.00 | Inulin | 9.99E-05 | (uncharacterised Ruminococcaceae) |
| Otu0031 | 1.35 | Inoculum | 4.47E-10 | *Guyana massiliensis* |
| Otu0032 | 1.60 | - |  | *Ruminococcus bromii* (93% similarity) |
| Otu0033 | 0.00 | Inulin | 0.001563 | *Clostridium ramosum* |
| Otu0035 | 1.11 | - |  | *Barnesiella intestinihominis* |
| Otu0036 | 0.05 | - |  | *Lactobacillus* spp. |
| Otu0037 | 0.63 | Inulin | 4.06E-05 | *Faecalibacterium prausnitzii* |
| Otu0038 | 0.91 | Inoculum | 0.008136 | (uncharacterised Ruminococcaceae) |
| Otu0040 | 0.01 | - |  | *Acidaminococcus intestini* |
| Otu0041 | 1.03 | - |  | *Eubacterium rectale* |

.

2. Salter SJ, Cox MJ, Turek EM, Calus ST, Cookson WO, Moffatt MF, et al. Reagent and laboratory contamination can critically impact sequence-based microbiome analyses. BMC Biol. 2014;12:1.
