# Supplementary material for: Modulation of the human gut microbiota by dietary fibres occurs at the species level
Source: BMC Biol. 2016 Jan 11;14:3. doi: 10.1186/s12915-015-0224-3 (PMC4709873; doi:10.1186/s12915-015-0224-3)
Supplement: Additional file 6: Figure S3. — Changes in the human faecal microbial community with inulin monitored by qPCR. (DOCX 932 kb) [file 12915_2015_224_MOESM6_ESM.docx]

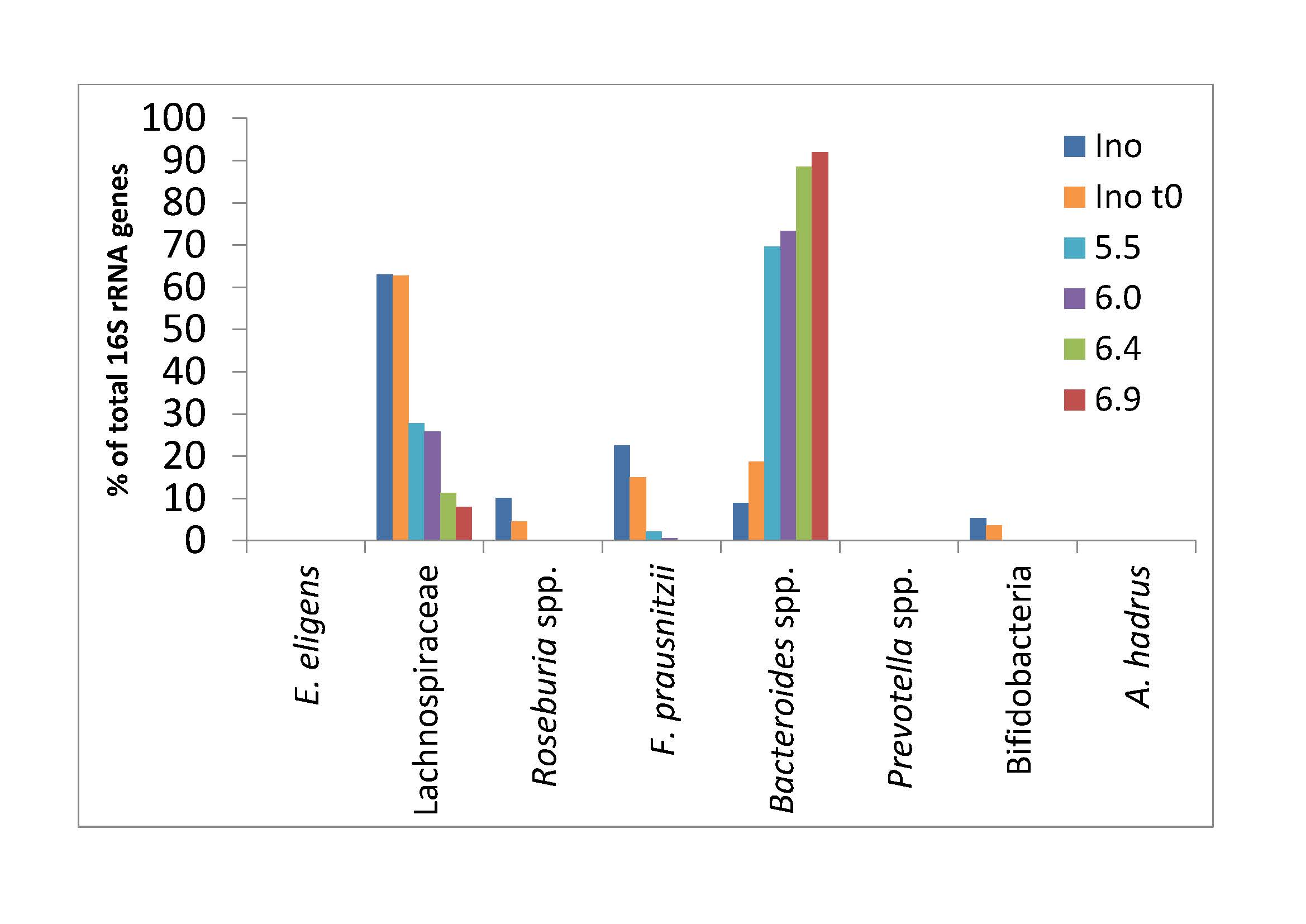

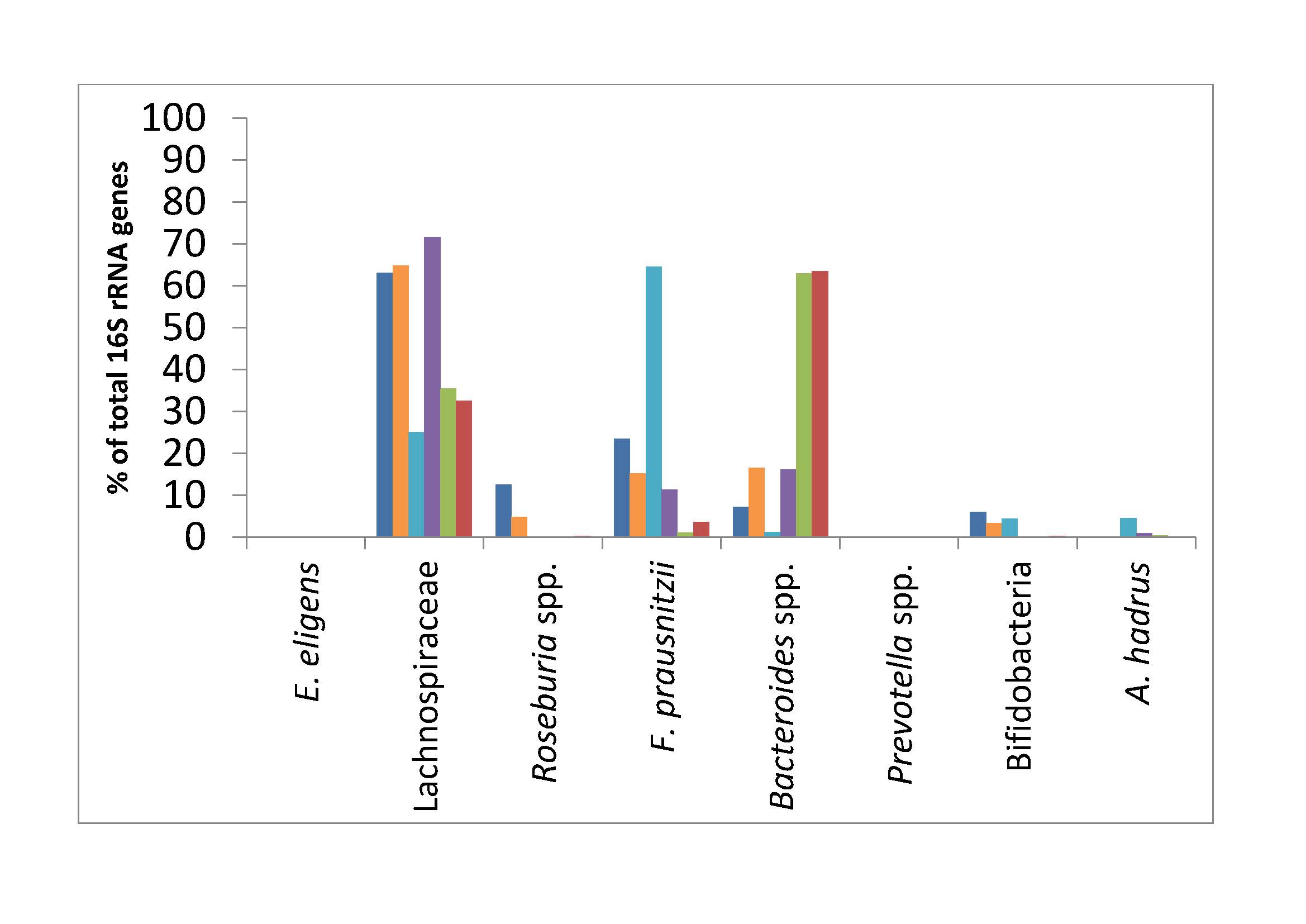

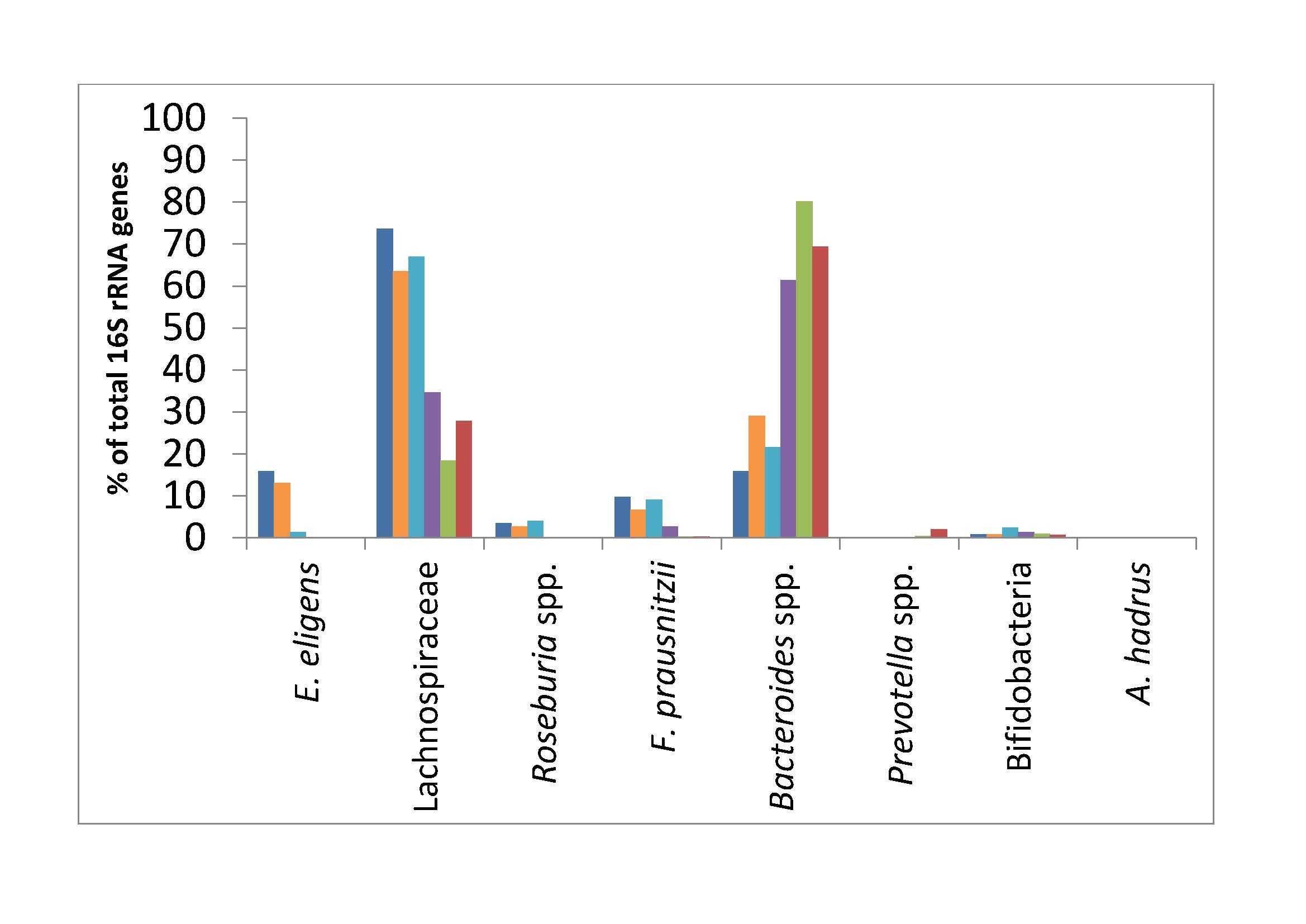

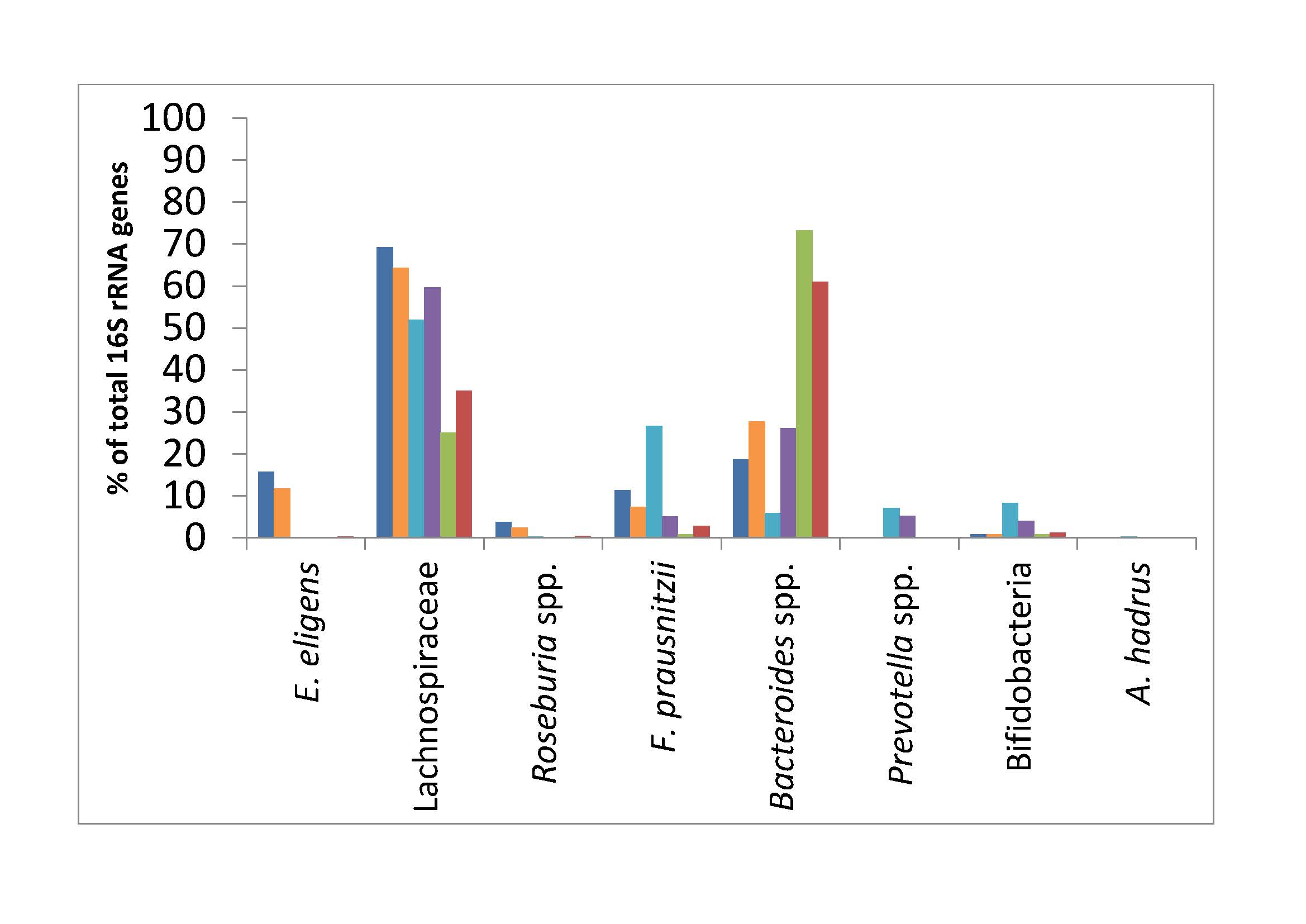

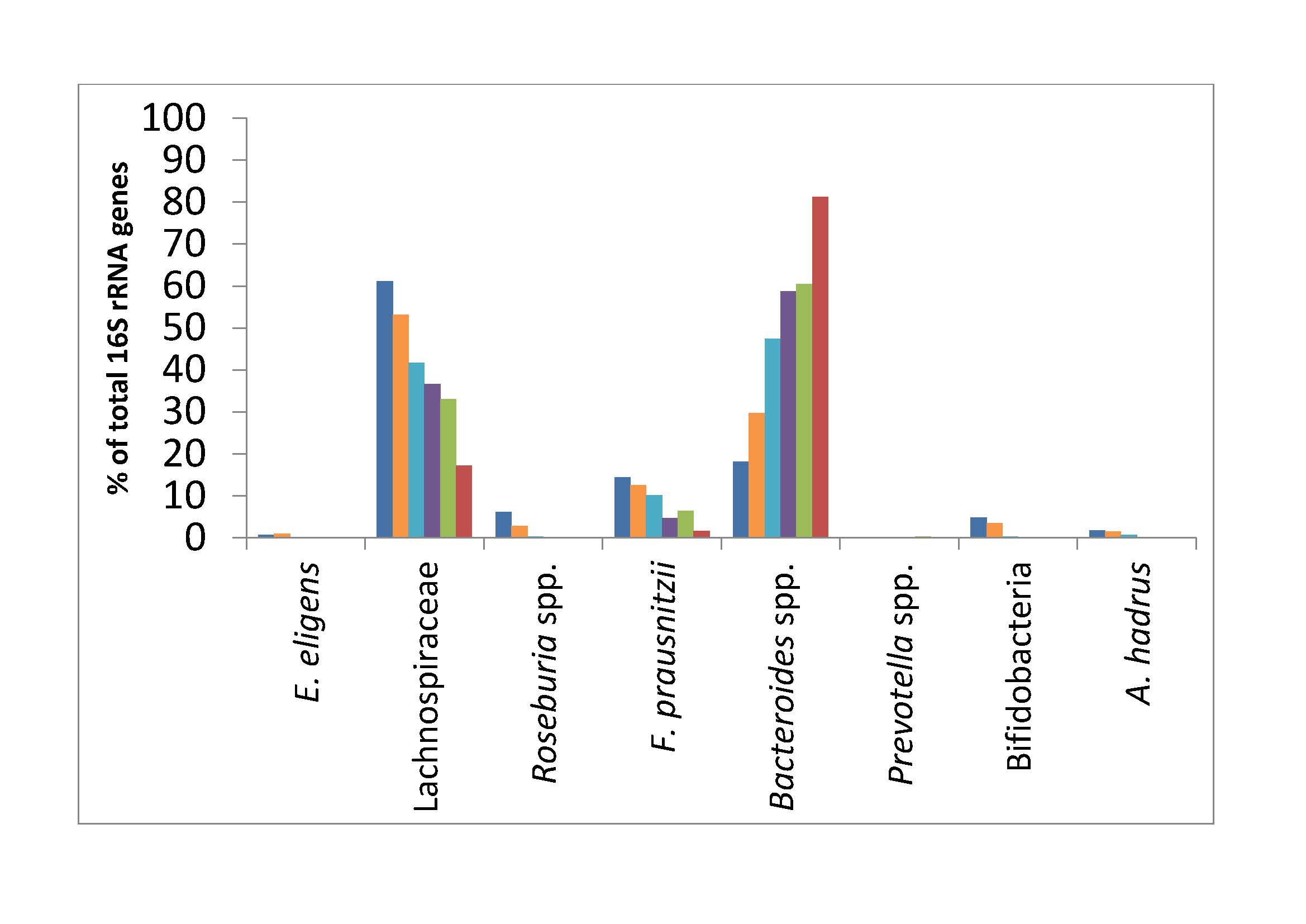

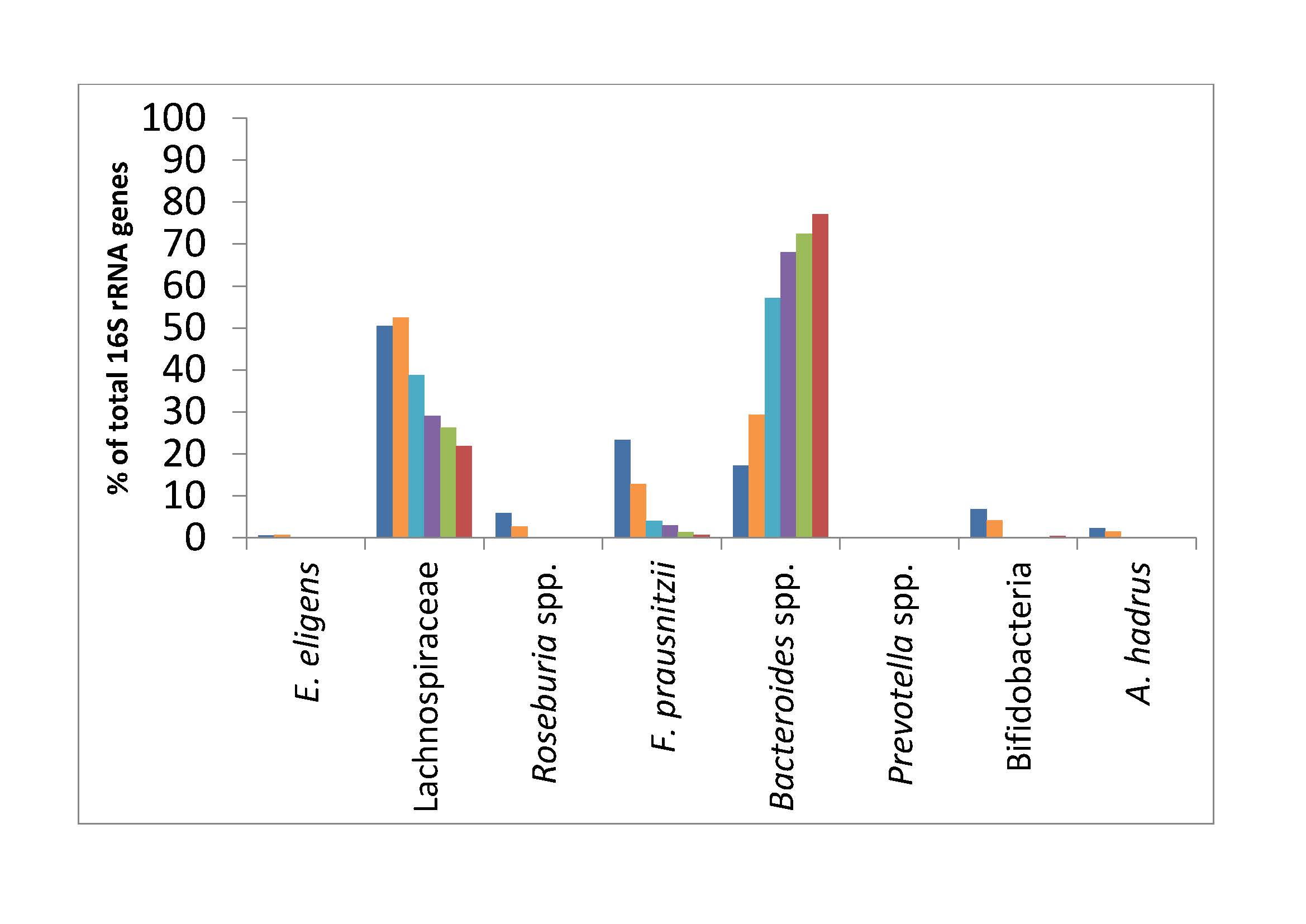


**F**

**E**

**D**

**C**

**B**

**A**

**Figure 3S**. **Changes in the human faecal microbial community with inulin monitored by qPCR.** Abundance for each bacterial group is expressed as a percentage, relative to the total bacteria detected (for primers used see Table S5). Separate parallel fermentor experiments were conducted with faecal inocula from three different donors (see Figure 1): Donor 1 fermentor 1 (A), Donor 1 fermentor 2 (B), Donor 2 fermentor 1 (C), Donor 2 fermentor 2 (D), Donor 3 fermentor 1 (E), Donor 3 fermentor 2 (F). Fermentor 1 was shifted in the sequence Ino (faecal slurry), Ino t0, pH 5.5, 6.0, 6.4, 6.9 and fermentor 2 in the sequence Ino (faecal slurry), Ino t0, pH 6.9, 6.4, 6.0, 5.5. *E. eligens* = *Eubacterium eligens*, *F. prausnitizii* = *Faecalibacterium prausnitzii*, *A. hadrus* = *Anaerostipes hadrus*. The results of 16S rRNA gene amplicon sequence analysis for the same samples are shown in Additional file 2: Table 1S and Figures 2-7.
