# Supplementary material for: Modulation of the human gut microbiota by dietary fibres occurs at the species level
Source: BMC Biol. 2016 Jan 11;14:3. doi: 10.1186/s12915-015-0224-3 (PMC4709873; doi:10.1186/s12915-015-0224-3)
Supplement: Additional file 7: Table S4. — Summary table of mean short chain fatty acid (SCFA) concentrations (mM) in pectin and inulin fermentors across all pH values. (DOCX 38 kb) [file 12915_2015_224_MOESM7_ESM.docx]

**Table 4S**. **Summary table of mean short chain fatty acids (SCFA) concentrations (mM) in pectin and inulin fermentors across all pH values.** Mean of data from three donors.

| **Pectin** | | | | |  | **Inulin** | | | | |
| --- | --- | --- | --- | --- | --- | --- | --- | --- | --- | --- |
| **F1** | **Formate** | **Acetate** | **Propionate** | **Butyrate** |  | **F1** | **Formate** | **Acetate** | **Propionate** | **Butyrate** |
| **5.5** | 1.53±1.80 | 30.39±4.63 | 5.06±2.34 | 7.19±2.17 |  | **5.5** | 1.50±0.89 | 21.48±7.09 | 6.38±2.10 | 6.91±1.98 |
| **6** | 1.51±1.66 | 31.85±13.40 | 7.91±5.15 | 6.10±4.31 |  | **6** | 1.49±1.22 | 18.64±10.24 | 8.80±4.69 | 7.50±5.48 |
| **6.4** | 2.13±2.87 | 45.03±17.00 | 9.80±5.15 | 6.94±2.84 |  | **6.4** | 1.67±0.88 | 21.52±8.88 | 11.02±3.77 | 8.66±6.23 |
| **6.9** | 1.88±1.11 | 51.63±29.80 | 12.18±8.08 | 8.34±5.75 |  | **6.9** | 1.23±1.38 | 22.32±5.76 | 11.80±2.51 | 7.87±3.72 |
|  |  |  |  |  |  |  |  |  |  |  |
| **F2** | **Formate** | **Acetate** | **Propionate** | **Butyrate** |  | **F2** | **Formate** | **Acetate** | **Propionate** | **Butyrate** |
| **5.5** | 3.06±1.78 | 26.60±10.46 | 4.47±2.07 | 5.32±2.35 |  | **5.5** | 1.02±1.92 | 12.60±7.06 | 8.78±7.22 | 9.34±6.14 |
| **6** | 2.72±3.26 | 45.38±18.42 | 10.67±7.41 | 10.47±6.37 |  | **6** | 0.41±1.62 | 18.74±7.02 | 13.45±3.87 | 8.40±5.74 |
| **6.4** | 2.18±2.80 | 50.74±29.94 | 13.05±11.00 | 11.10±7.98 |  | **6.4** | 0.64±1.27 | 16.41±4.86 | 10.62±2.68 | 5.01±2.39 |
| **6.9** | 1.36±1.66 | 42.88±18.85 | 9.87±6.68 | 9.04±5.21 |  | **6.9** | 0.42±1.61 | 21.15±7.01 | 9.80±2.98 | 5.76±3.04 |
