# Supplementary material for: Modulation of the human gut microbiota by dietary fibres occurs at the species level
Source: BMC Biol. 2016 Jan 11;14:3. doi: 10.1186/s12915-015-0224-3 (PMC4709873; doi:10.1186/s12915-015-0224-3)
Supplement: Additional file 8: Figure S4. — Correlation of metabolite concentrations with key propionate and butyrate producers as determined by qPCR in both apple pectin and inulin fermentors. (DOCX 43 kb) [file 12915_2015_224_MOESM8_ESM.docx]

**Figure 4S**. **Correlation of metabolite concentrations with key propionate and butyrate producers as determined by qPCR in both apple pectin and inulin fermentors**. Fig. 4S (A) shows the proportional abundance (as a percentage) of propionate plotted against the proportional abundance of *Bacteroides* spp. and *Prevotella* spp. and (B) the percentage butyrate against the dominant butyrate producers *Faecalibacterium prausnitzii* and *Roseburia* species. The data are the mean from three donors.
