# Supplementary material for: Modulation of the human gut microbiota by dietary fibres occurs at the species level
Source: BMC Biol. 2016 Jan 11;14:3. doi: 10.1186/s12915-015-0224-3 (PMC4709873; doi:10.1186/s12915-015-0224-3)
Supplement: Additional file 9: Table S5. — List of qPCR primers used in this study including information on primer target, sequence, annealing temperature and references. (DOCX 39 kb) [file 12915_2015_224_MOESM9_ESM.docx]

**Table 5S**. **List of qPCR primers used in this study including information on primer target, sequence, annealing temperature and references**. (*New primers were designed as described previously [3] by inspection of alignments downloaded from the Ribosomal Database Project (RDP [4]) and by RDP Probe match analysis. Primers were validated against 16S rRNA gene amplicons of 37 strains of bacteria that included five Bacteroidetes and 18 Lachnospiraceae representatives).

| **Target** | **Name** | **Sequence** | **Primer annealing temp (C)** | **Reference** |
| --- | --- | --- | --- | --- |
| Universal | UniF | GTGSTGCAYGGYYGTCGTCA | 60 | Fuller et al., 2007[5] |
|  | UniR | ACGTCRTCCMCNCCTTCCTC |  |  |
| *Faecalibacterium prausnitzii* | FPR-2F | GGAGGAAGAAGGTCTTCGG | 60 | Ramirez-Farias et al., 2009[3] |
|  | Fprau645R | AATTCCGCCTACCTCTGCACT |  |  |
| *Bifidobacterium* spp. | BifF | TCGCGTCYGGTGTGAAAG | 60 | Fuller et al., 2007[5] |
|  | g-Bifid-R | GGTGTTCTTCCCGATATCTACA |  |  |
| Clostridial Cluster XIVa (Lachnospiraceae) | Erec482F | CGGTACCTGACTAAGAAGC | 60 | Ramirez-Farias et al., 2009[3] |
|  | Erec870R | AGTTTYATTCTTGCGAACG |  |  |
| *Roseburia* spp. & *E. rectale* | RrecF | GCGGTRCGGCAAGTCTGA | 63 | Ramirez-Farias et al., 2009[3] |
|  | Rrec630mR | CCTCCGACACTCTAGTMCGAC |  |  |
| *Bacteroides* spp | g-Bfra-R-Fmod | GCTCAACCKTAAAATTGCAGTTG | 63 | Matsuki et al., 2002[6] modified, Bartosch et al., 2004[7] modified |
|  | Bac708Rmod | GCAATCGGRGTTCTTCGTG |  |  |
| *Prevotella* spp. | g-Prevo-Fmod | CRCRCRGTAAACGATGGATG | 65 | Matsuki et al., 2002[6] modified, Wood et al., 1998[8] modified |
|  | BacPreRmod | TTGAGTTTCACCGTTGCCGG |  |  |
| *Eubacterium eligens* | EelF | CGCACAATGTTGCATGACAT | 63 | This study, Rinttilä et al., 2004[9] modified |
|  | EelR | CTTAGTCAGGTACCGTCACTA |  |  |
| *Anaerostipes hadrus* | SSC2F2 | CTTTAGTAGCCAGCATATAAGG | 60 | Louis et al 2010[10] |
|  | SSC2R | TTGCTCACTCTCACGAGGCT |  |  |

**Additional References**

3. Ramirez Farias C, Slezak K, Fuller Z, Duncan A, Holtrop G, Louis P. Effect of inulin on the human gut microbiota: stimulation of Bifidobacterium adolescentis and Faecalibacterium prausnitzii. Br J Nutr. 2009;101:4:541-50.

4. Cole JR, Wang Q, Fish JA, Chai B, McGarrell DM, Sun Y, et al. Ribosomal Database Project: Data and tools for high throughput rRNA analysis. Nucleic Acids Res. 2014;42:D1:D633-42.

5. Fuller Z, Louis P, Mihajlovski A, Rungaparnestry V, Ratcliffe B, Duncan AJ. Influence of cabbage processing methods and prebiotic manipulation of colonic microflora on glucosinolate breakdown in man. Br J Nutr. 2007;98:2:364-72.

6. Matsuki T, Watanabe K, Fujimoto J, Miyamoto Y, Takada T, Matsumoto K, et al. Development of 16S rRNA-gene-targeted group-specific primers for the detection and identification of predominant bacteria in human feces. Appl Environ Microbiol. 2002;68:11:5445-51.

7. Bartosch S, Fite A, Macfarlane G, McMurdo M. Characterization of bacterial communities in feces from healthy elderly volunteers and hospitalized elderly patients by using real-time PCR and effects of antibiotic treatment on the fecal microbiota. Appl Environ Microbiol. 2004;70:6:3575-81.

8. Wood J, Scott K, Avgustin G, Newbold C, Flint H. Estimation of the relative abundance of different Bacteroides and Prevotella ribotypes in gut samples by restriction enzyme profiling of PCR-amplified 16S rRNA gene sequences. Appl Environ Microbiol. 1998;64:10:3683-9.

9. Rinttila T, Kassinen A, Malinen E, Krogius L, Palva A. Development of an extensive set of 16S rDNA-targeted primers for quantification of pathogenic and indigenous bacteria in faecal samples by real-time PCR. J Appl Microbiol. 2004;97:6:1166-77.

10. Louis P, Young P, Holtrop G, Flint HJ. Diversity of human colonic butyrate-producing bacteria revealed by analysis of the butyryl-CoA:acetate CoA-transferase gene. Environ Microbiol. 2010;12:2:304-14.
